# Maternal well-being and its association to risk of developmental problems in children at school entry

**DOI:** 10.1186/1471-2431-10-19

**Published:** 2010-03-25

**Authors:** Suzanne C Tough, Jodi E Siever, Karen Benzies, Shirley Leew, David W Johnston

**Affiliations:** 1 Department of Paediatrics, Faculty of Medicine, University of Calgary, Calgary, Alberta, Canada; 2 Department of Community Health Sciences, Faculty of Medicine, University of Calgary, Calgary, Alberta, Canada; 3 Public Health Innovation and Decision Support, Population and Public Health, Alberta Health Services, Calgary, Alberta, Canada; 4 Faculty of Nursing, University of Calgary, Calgary, Alberta, Canada; 5 Decision Support Research Team, Alberta Health Services, Calgary, Alberta, Canada; 6 Behavioural Research Unit, Alberta Children's Hospital, Calgary, Alberta, Canada

## Abstract

**Background:**

Children at highest risk of developmental problems benefit from early identification and intervention. Investigating factors affecting child development at the time of transition to school may reveal opportunities to tailor early intervention programs for the greatest effectiveness, social benefit and economic gain. The primary objective of this study was to identify child and maternal factors associated with children who screened at risk of developmental problems at school entry.

**Methods:**

An existing cohort of 791 mothers who had been followed since early pregnancy was mailed a questionnaire when the children were aged four to six years. The questionnaire included a screening tool for developmental problems, an assessment of the child's social competence, health care utilization and referrals, and maternal factors, including physical health, mental health, social support, parenting morale and sense of competence, and parenting support/resources.

**Results:**

Of the 491 mothers (62%) who responded, 15% had children who were screened at high risk of developmental problems. Based on a logistic regression model, independent predictors of screening at high risk for developmental problems at age 5 were male gender (OR: 2.3; 95% CI: 1.3, 4.1), maternal history of abuse at pregnancy (OR: 2.4; 95% CI: 1.3, 4.4), and poor parenting morale when the child was 3 years old (OR: 3.9; 95% CI: 2.1, 7.3). A child with all of these risk factors had a 35% predicted probability of screening at high risk of developmental problems, which was reduced to 13% if maternal factors were favourable.

**Conclusions:**

Risk factors for developmental problems at school entry are related to maternal well being and history of abuse, which can be identified in the prenatal period or when children are preschool age.

## Background

In the early years, unique opportunities exist to influence children's development trajectories and their families [1-4]. Birth to five years is a critical time in development when language, cognitive, emotional, social, behavioural and physical skills become the basis for the scaffolding of new skills throughout further experiences and education into adult life [4-6]. Childhood development has a demonstrable influence on long-term outcomes, affecting academic performance, health, coping skills and successes throughout the life course [2,5,6]. Evidence indicates that children at highest risk of developmental problems benefit from early identification and intervention [1-4]. Early interventions not only enhance the well-being of children and the resources of families but also benefit society by preventing and minimizing developmental problems and their costly consequences [1,3,7-11].

With an estimated 780 million young children around the world affected by an intellectual disability, the impact of developmental problems in society is widespread [4]. Understanding who is at risk in our community, and who would benefit from early intervention, allows for optimal allocation of limited resources. Investigating factors affecting child development at the time of school entry may reveal opportunities to tailor early intervention programs for the greatest effectiveness, social benefit and economic gain.

It is well-known that sociodemographic characteristics of the family, such as low income and less maternal education, put children at higher risk for poor outcomes [10,12-14]. Recent evidence also suggests that, neighbourhood factors, such as neighbourhood income and cohesion, may also impact child development [15]. Males are at increased risk for developmental problems [14], and exposure to substances in utero impacts birth outcomes and child development [16,17].

Maternal well-being and parenting have also been associated with child development. Harsh or inconsistent parenting approaches as well as maternal stress and anxiety have been associated with child behavioural outcomes [9,18-21]. A maternal history of abuse may also be associated with infant temperament and child behaviour [22,23]. Post-partum depression has been associated with poor cognitive and emotional development in infants, particularly among males, and these effects may persist into early childhood [14,19,24-28]. Predictable, appropriate responses to infants from adults are critical to optimal child development, and poor maternal mental health, in particular, can interfere with the creation of early infant-maternal social interactions that are stimulating and supportive [18-20]. Furthermore, parental models of attachment, family dysfunction, and family structure (e.g. lone parenting) have also been associated with parent-child interactions and behaviour problems in children [9,12,13,29]. Lack of maternal social support may also adversely impact child cognitive and behavioural development, most notably when mothers are dealing with depression or have a history of abuse [22,30,31]. The relationships among these maternal and family factors are complex as some factors may mediate the impact of others [22,29]. Furthermore, risk for delay and poor outcomes are cumulative such that children exposed to a greater number of risks have poorer outcomes than children exposed to less risk [13].

The study described in this paper follows up a cohort of mothers and children that have been studied on two previous occasions--during the perinatal period and when children were three years of age. Based in Calgary, an urban centre of over 1 million people in Alberta, Canada, the original Community Perinatal Care Study was a randomized controlled trial (RCT) of three types of prenatal care [32]. The results showed that additional prenatal support from nurses and home visitors could increase the use of community based resources and the access of pregnancy related information. The additional support, however, did not translate into changes in alcohol and tobacco use, post-partum depression or birth outcomes [32].

When the children in the Community Perinatal Care Study were followed up in a study at three years of age, the participants constituted a demographically low risk sample with regards to maternal education and income [14]. Of note, 73% of all Canadian families with kids under six years of age have a household income greater than or equal to $40,000 [33]. The follow-up study at three years found that 11% of children screened at high risk of developmental problems [14]. Type of prenatal care received in the RCT did not have an impact on the child's risk for developmental problems at three years of age [14]. However, a male child who had a history of ear infections and was living in a low income environment with a mother with poor mental health (defined as a history of postpartum depression, abuse, and/or lack of contentment during pregnancy), had a predicted probability of 53% of having developmental problems at three years of age [14]. With all other variables held constant, this risk was reduced to 19% if the mother reported good mental health during the prenatal and early postpartum period, suggesting a meaningful potential benefit of interventions designed to address maternal mental health and well being [14].

The current study was conducted to answer questions about the health and development of a community based sample of children as they became of school age. The objectives of this study were to:

• identify the child and maternal factors associated with children who screened at risk of developmental and behavioural/emotional problems at five years of age,

• develop a model that predicts risk for developmental and behavioural/emotional problems at five years of age based on historical factors, and

• determine if the factors related to high risk for developmental problems at three years of age persisted as these children entered school.

For the purposes of this paper, developmental problems refer to a child's difficulties affecting development in any one, any combination, or all (i.e., global dysfunction) of the following domains: cognitive, social, language, motor, academics.

## Methods

### Participants

Participants in this study are part of a well-established cohort of women followed from early pregnancy to the time their children entered school. The original Community Perinatal Care (CPC) study began recruiting participants in 2000 through three family physician maternity clinics in the city of Calgary, inviting women at low medical risk to contribute to the study as they were seeking to begin prenatal care. In a randomized controlled trial, 1,737 women were assigned to three study groups, one receiving the current standard of prenatal care, a second receiving the same with the addition of consultation with a nurse trained in prenatal care, and the third receiving standard care, consultation with a trained nurse as well as home visits from a paraprofessional trained in non-medical prenatal care. Three computer-assisted telephone interviews followed over the course of the pregnancies in the first trimester, at 32-34 weeks gestation and eight weeks after delivery, and the women were also invited to participate in future research. Mothers who agreed to participate further formed the participant base for the first follow up study when their children were three years of age. With a focus on parenting and child development, the follow-up study at three years collected information from 791 women using telephone questionnaires and built on information from the cohort established during the original study. Detailed methods and full results from the CPC study and the first follow up study when children were three years of age are reported elsewhere [14,32]. Two years later, when the children were aged four to six years, the participants from the follow up study when children were three years of age formed the cohort for this second follow up study and received a mailed questionnaire. Mothers were excluded if they could not speak English and refused participation in the first follow up study.

### Questionnaire

This questionnaire followed similar themes as were addressed in the follow up study when children were three years of age. In addition to demographic data, specific questions addressed the child's development, social competence, health care utilization, and referrals. Other questions addressed maternal physical health, emotional health, and social support as well as parenting, including morale, sense of competence, and supports and resources (see Additional file 1).

The questionnaire included six standardized measurement scales. One of these was the Parents' Evaluation of Development Status (PEDS), a 10 item parent-report screening measure to facilitate detection of risk for developmental and behaviour/emotional problems [34]. This was our primary outcome measure. Using the PEDS, parents report their concerns in 10 areas: global/cognitive, expressive language, receptive language, fine motor, gross motor, behaviour, social-emotional, self-help, school, and other issues (typically medical or sensory) [35]. The PEDS screens children by level of risk for developmental disabilities and behavioural/emotional problems (i.e. assigns PEDS 'paths') with a sensitivity and specificity that ranges between 70% and 80% [34]. At 4 1/2 to 6 years of age, concerns reported by parents in the global/cognitive, expressive language, receptive language, fine motor, gross motor, school, and other areas are predictive of developmental disabilities [35,36]. Scoring of the PEDS categorizes children into one of fives paths:

• Path A: high risk for developmental problems

• Path B: moderate risk for developmental problems

• Path C: low risk for developmental problems but elevated risk for behavioural/emotional problems

• Path D: parental communication difficulties

• Path E: low risk of developmental and behavioural/emotional problems.

Also included were the Child Social Competence Scale for parents to assess pro-social behaviour, communication and self-control in the child, the SF-8 to assess maternal physical and mental health, and the Medical Outcomes Study Social Support Scale to assess maternal social support [37-39]. To examine parenting further, the Parenting Sense of Competence Scale and the Parenting Morale Index were included as well [40,41]. The Parenting Morale Index measures "parenting zeal, enthusiasm, and capability to endure hardship" by measuring the frequency of ten emotional states of parents, and the internal consistency of this relatively new scale is strong (Cronbach's alpha = 0.84) [41]. Table 1 contains detailed information on the standardized measurement scales that were part of the questionnaire. The entire questionnaire was pilot tested with 10 mothers for length, flow, and comprehension and was revised as per comments and consultations.

**Table 1:** Detailed description of standardized measurement scales used in the study

Scale	Description
Parents' Evaluation of Development Status (PEDS)	The PEDS is a parent-reported screening measure to facilitate detection of developmental and behavioural/emotional problems [34]. The scale contains 10 items: global/cognitive, expressive language, receptive language, fine motor, gross motor, behaviour, social-emotional, self-help, school, and other issues (typically medical or sensory) [35]. Scoring of the PEDS categorizes children into one of five paths, depending on their level of risk for developmental and behavioural/emotional problems.

Child Social Competence Scale	Using a 5-point Likert scale, the Social Competence Scale - Parent Version assesses a child's prosocial behaviours, communication skills, and self control. The scale has two subscales: prosocial/communication skills and emotional regulation skills. The scale has 12 items that each refer to a behaviour that a child may exhibit in a social setting. The parent reports how well each statement describes the child. Examples of statements include: "Your child can give suggestions and opinions without being bossy" and "Your child can calm down when excited or 'all wound up"' [37].

SF-8	The SF-8 Health Survey is a widely used generic multipurpose short-form (SF) survey of health status with sub-scales for mental and physical health. The scale has eight items to measure eight domains of health: physical functioning, role-physical, bodily pain, general health, vitality, social functioning, role-emotional, and mental health [38].

Medical Outcomes Study Social Support Scale	Using a 5-point Likert scale, the Medical Outcomes Study Social Support Scale measures functional social support according to four subscales and also provides an overall measure of support. The scale has 19 items, and respondents indicate how often each kind of support was available to them if they need it. The four subscales are: emotional/informational ("the expression of positive affect, empathetic understanding, the encouragement of expression of feelings" and "the offering of advice, information, guidance, or feedback"), tangible ("the provision of material aid or behavioral assistance"), affection ("expressions of love and affection"), and positive social interaction ("other people to do fun things with you") [39].

Parenting Sense of Competence Scale	Using a 6-point Likert scale, the Parenting Sense of Competence Scale assesses the degree to which a parent feels competent and confident in handling a child's problems and the degree of satisfaction they associate with parenting. The scale contains 16 items. Examples of items include: "Being a parent is manageable, and any problems are easily solved" and "Sometimes I feel like I'm not getting anything done" [40].

Parenting Morale Index (PMI)	Using a 5-point Likert scale, the PMI measures how frequently parents experience ten emotional states (optimistic, worried, contented, frustrated, satisfied, happy, stressed, lonely, exhausted, and guilty). The scale contains 10 items [41].

Questionnaires were mailed to the 791 respondents from the first follow up study with a cover sheet detailing their voluntary participation, confidentiality and the links that would be made to previously collected data. The questionnaire took approximately 15-20 minutes to complete. It was requested that the questionnaire be mailed back within two weeks, after which time telephone calls were made to offer an alternative response mode. Women were considered unreachable if they could not be contacted by telephone due to a change in phone number and alternate contacts could not be reached. Data collection began in August 2007 and was completed by January 2008. Questionnaires were scanned and verified in Teleform, an electronic data capture and management system [42]. Ethics approval was granted to the study from the Conjoint Health Research Ethics Board at the University of Calgary.

### Analysis

The data collected through the questionnaires were linked to data from the original CPC study as well as to the first follow up study using unique study identification numbers. This resulted in data for mothers at five time points from the first trimester to 5 years post partum. Data were analyzed using the statistical package Stata/SE Version 10.0 [43]. The data analysis included descriptive methods for categorical data as well as bivariate and multivariate methods. Based on the PEDS scoring, children were categorized into one of the five PEDS paths (Path A, B, C, D, or E), which indicated the level of risk for developmental and behavioural/emotional problems as noted above. The variables based on the Child Social Competence Scale, SF-8, Medical Outcomes Study Social Support Scale, Parenting Sense of Competence Scale, and Parenting Morale Index were dichotomized at the value closest to the 25^th ^percentile. Scores equal to or below this cutoff were categorized as high risk while scores above the cutoff were categorized as low risk. During the prenatal period, women had been asked about abuse in the following way: "Abuse can take many forms: physical, emotional (including psychological or verbal), sexual, financial (e.g. withholding or controlling money) or neglect. We ask all participants in this study about abuse in their lives. Have you ever been physically abused, emotionally abused, sexually abused, financially abused, or neglected?" Women who indicated they had been abused in any form were considered to have a history of abuse.

Bivariate analysis, using Fisher's exact test, was undertaken to compare PEDS paths on child and maternal characteristics. Statistical significance was set at p < 0.05. A multinomial logistic regression model was constructed to explore the relationship between risk of developmental problems and historical factors associated with this risk, yielding odds ratios and 95% confidence intervals. Variables in the bivariate analysis were considered eligible for inclusion in the regression modeling if they were significant at p ≤ 0.10, or if prior evidence or theoretical considerations supported their inclusion. Characteristics of the child were entered into the regression model first (e.g. gender, ear infections prior to age two), then maternal measures (e.g. parenting morale, social support, physical health, mental health), and lastly socio-demographic measures. In addition, variables were entered into the model from past to present (e.g. history of abuse reported at pregnancy was entered before parenting morale measured at 3 years post partum). When measurement scales had subscales, the overall index was used in the regression model. Selected predicted probabilities for screening in each PEDS path were also calculated. Although the current study was a follow-up of a cohort over a period of over 5 years, data on child development were only collected at two points, thus limiting our ability to conduct a longitudinal analysis of the child development data.

## Results

### Participation and characteristics

**Figure 1:**
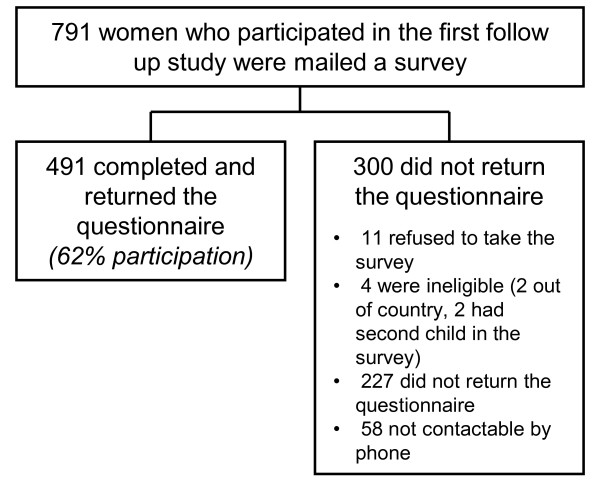
**Study flowchart mapping eligibility, recruitment, and completion of mothers who participated in the study**.

Of the 791 respondents in the three year follow up study, 491 returned questionnaires when their children were at school entry, resulting in a 62% response rate (Figure 1). The majority of mothers who participated were married or with a partner, educated at a post-secondary level and had a household income over $40,000 per year (Table 2). Almost half reported being a homemaker. The average age of the children was five years, and about half were male. The majority of previous referrals had been to speech and language pathologists. Thirty-two percent of women reported a history of abuse when asked during pregnancy. Of the total sample, 61 women reported physical abuse (12.4%), 110 emotional abuse (22.4%), 65 sexual abuse (13.2%), 20 financial abuse (4.1%), and 16 neglect (3.3%). Mothers who were lost to follow up and did not participate in this study were more likely to have reported the following in the previous study: smoking, not having attended parenting classes or been pregnant again, poor physical health ratings, incomes less than $40,000 per year, less than a high school education, being single or divorced and/or being less than 25 years of age (all p < 0.05, data not tabled). There was no association between the type of prenatal care women had received during the RCT and the risk of developmental problems in their children at school age.

**Table 2:** Characteristics of mothers and children who participated in the follow up study

Characteristic	N = 491	
**Mothers**	**Mean**	**sd**

Age	36.3	4.4

	**n**	**%**

Marital Status		
Married/Commonlaw	473	95.0
Divorced/Separated	17	3.4
Single/Widowed	8	1.6

Main Activity		
Working at a job or business (full/part-time)	208	42.6
Homemaker	226	46.2
Looking for work	2	0.4
Paid maternity leave	28	5.7
Student	5	1.0
Other	20	4.1

Household Income		
≤$39,999	24	4.9
$40,000-$79,999	124	25.5
$80,000-$119,999	154	31.6
≥$120,000	149	30.6
Prefer not to answer	36	7.4

Education		
Less than high school	0	0.0
High school	80	16.4
College/trade	158	32.3
University	197	40.3
Post graduate studies	54	11.0

Moved once or more in the past year	93	19.2

**Children**	**Mean**	**sd**

Age	5.0	0.6

	**n**	**%**

Male	240	48.9

Child has had routine health exam in the past year	394	80.7

Child has had his/her vision or eyes checked in the past year	261	53.8

Child has had his/her hearing tested in the past year	58	12.0

Child has seen a dentist in the past year	436	89.0

In the past year, child has been referred to:		
Early intervention program Speech and language pathologist	6 57	1.2 11.6
Child developmental paediatrician	14	2.9
Psychologist	11	2.2
Physiotherapist	5	1.0
Dietician	5	1.0

Note: Denominator varies due to missing data on some survey items.

### Children's development and behaviour

Analysis of the PEDS revealed that 15% (n = 72) of the children screened at the highest risk of developmental problems (Path A). Of these 72, 53% (n = 38) had at least one referral for further assessment or intervention since birth with 33% (n = 24) referred within the last year. Thirty-one percent (n = 153) of the children screened at moderate risk of developmental problems (Path B). Nineteen percent (n = 93) of the children screened in Path C which suggests low risk for developmental problems but elevated risk for behavioural/emotional problems. The remaining 35% (n = 172) of children were screened to Path E which suggests a low risk of either developmental or behavioural/emotional problems. None of the respondents were screened in Path D for parental communication difficulties. Of concerns reported by parents that are predictive of developmental problems, the most commonly reported was expressive language. Of concerns that are not predictive of developmental problems, behavioural and social-emotional concerns were reported most often by the mothers.

### Factors associated with risk of developmental problems

An increased risk for developmental problems was associated with lower social competence scores for children (p < 0.001) (Table 3). Of the children at high risk for developmental problems or elevated risk for behavioural/emotional problems (Path A or C), more than 40% also scored in the lowest quartile of overall social competence. Male children were also significantly more likely to screen at risk of developmental problems (Table 3).

**Table 3:** Maternal and child characteristics, by PEDS path of child

Scale	Path A (high risk for developmental problems) N = 72 n (%)	Path B (moderate risk for developmental problems) N = 153 n (%)	Path C (elevated risk for behavioural/emotional problems) N = 93 n (%)	Path E (low risk for problems) N = 172 n (%)	Total N = 490 n (%)	p-value
**Child Factors**						

Male	45 (63)	82 (54)	43 (46)	70 (41)	240 (49)	0.009

Ear infections prior to age 2	29 (41)	52 (35)	37 (40)	56 (33)	174 (36)	0.503

Poor overall social competence*	32 (45)	40 (26)	38 (41)	30 (17)	140 (29)	<0.001

Poor prosocial/communication skills*	30 (42)	40 (26)	32 (34)	21 (12)	123 (25)	<0.001

Poor emotional regulation skills*	35 (49)	44 (29)	41 (44)	35 (20)	155 (32)	<0.001

**Current Maternal Factors**						

SF-8						

Poor physical health*	25 (36)	39 (26)	22 (24)	35 (21)	121 (25)	0.114

Poor mental health*	26 (37)	45 (30)	21 (23)	29 (17)	121 (25)	0.004

Social Support						

Poor emotional/informational support*	27 (40)	34 (23)	29 (32)	30 (18)	120 (25)	0.003

Poor tangible support*	28 (41)	45 (30)	29 (31)	44 (26)	146 (30)	0.139

Poor positive interaction*	32 (47)	45 (30)	28 (30)	38 (22)	143 (30)	0.003

Poor affection*	30 (43)	34 (22)	26 (28)	37 (22)	127 (26)	0.006

Poor overall support index*	31 (46)	35 (24)	27 (30)	32 (19)	125 (27)	<0.001

Parenting Sense of Competence						

Low efficacy*	27 (40)	37 (25)	27 (29)	35 (21)	126 (27)	0.033

Low satisfaction*	28 (41)	36 (24)	29 (32)	31 (18)	124 (26)	0.002

Poor parenting morale*	31 (46)	43 (28)	30 (33)	33 (20)	137 (28)	0.001

**Historical Maternal Factors**						

Poor parenting morale at 3 years post partum	31 (43)	40 (26)	25 (27)	28 (16)	124 (25)	<0.001

Poor physical health at 3 years	24 (33)	34 (22)	21 (23)	27 (16)	106 (22)	0.024

Poor social support at 3 years post partum	12 (17)	15 (10)	10 (11)	15 (9)	52 (11)	0.324

Depression for 2 or more weeks post partum	32 (44)	52 (34)	32 (34)	52 (30)	168 (34)	0.212

Poor social support during pregnancy	17 (24)	32 (21)	23 (25)	22 (13)	94 (19)	0.045

Low score on positive feelings during pregnancy	21 (29)	46 (30)	33 (35)	44 (25)	144 (29)	0.406

History of abuse, at pregnancy	30 (42)	56 (37)	33 (35)	38 (22)	157 (32)	0.004

**Maternal Socio-Demographic Factors**						

Income <$80,000	29 (40)	48 (31)	26 (28)	45 (26)	158 (30)	0.169

No post-secondary education	13 (18)	24 (16)	17 (18)	26 (15)	80 (16)	0.881

No marriage or common-law partner	8 (11)	5 (3)	5 (5)	7 (4)	25 (5)	0.102

*Children or mothers scoring in the lowest quartile of these measures.

Note: Denominator varies due to missing data on some survey items. The denominator is not 491 in this table because one mother completed only half of the PEDS survey as her questionnaire was missing a page.

The children of mothers who reported poor mental health and low social support were significantly more likely to screen at high risk for developmental problems (Path A) (Table 3). Poor mental health was reported by 37% of mothers with children in Path A but by only 17% of mothers with children in Path E (p = 0.004) (Table 3). Of the mothers with children in Path A, 46% scored in the lowest quartile for current social support while just 19% of mothers with children in Path E scored in the same quartile (p < 0.001) (Table 3). Parenting sense of competence and morale were also associated with a high risk for developmental problems (Table 3). Forty percent of mothers with children in Path A scored in the lowest quartile of parenting efficacy and satisfaction with parenting (p < 0.035) compared to 20% of mothers with children in Path E, and 32% of mothers in Path A compared to 7% of mothers in Path E scored in the lowest quartile for parenting morale (p < 0.001) (Table 3).

Children who were screened at risk for developmental problems were significantly more likely to have mothers who, during pregnancy, had reported a history of abuse and poor social support (p < 0.05) (Table 3). They were also more likely to have mothers who reported poor physical health and poor parenting morale when their child was three years (p < 0.05) (Table 3). In the bivariate analysis, no significant differences were found in income, education or marital status between Paths.

### Key predictors of screening for high risk of developmental problems

Multinomial logistic regression analysis revealed that the most significant predictors of having a child who was screened at high risk (Path A) compared to low risk of developmental problems included a child who was male, a mother who had reported a history of abuse while pregnant and poor parenting morale when her child was three years old (Table 4). Based on the logistic regression model, a boy, whose mother had a history of abuse and poor parenting morale, had a 35% predicted probability of screening at high risk of developmental problems (Path A) (Table 5). However, a boy, whose mother had no history of abuse and a history of good parenting morale, had a 13% predicted probability of screening at high risk of developmental problems. Similarly, the predicted probability of screening at high risk for developmental problems for a girl, whose mother had a history of abuse and poor parenting morale, was 24%. A girl, whose mother had no history of abuse and a history of good parenting morale, had a 7% probability of screening at high risk for developmental problems (Table 5).

**Table 4:** Key predictors for scoring at risk of developmental problems on the PEDS, N = 490

Variable	Unadjusted Odds Ratio (95% C.I.)	Adjusted Odds Ratio (95% C.I.)	p-value
**Path A (high risk for developmental problems)**			

**Child Characteristics**			

Male	2.4 (1.4, 4.3)	2.3 (1.3, 4.1)	0.005

**Maternal Characteristics**			

Poor parenting morale, at 3 years post partum	3.9 (2.1, 7.2)	3.9 (2.1, 7.3)	<0.001

History of abuse, at pregnancy	2.5 (1.4, 4.6)	2.4 (1.3, 4.4)	0.006

**Path B (moderate risk for developmental problems)**			

**Child Characteristics**			

Male	1.7 (1.1, 2.6)	1.6 (1.0, 2.5)	0.033

**Maternal Characteristics**			

Poor parenting morale, at 3 years post partum	1.8 (1.1, 3.1)	1.8 (1.0, 3.1)	0.034

History of abuse, at pregnancy	2.0 (1.2, 3.3)	2.0 (1.2, 3.2)	0.007

**Path C (elevated risk for behavioral and/or behavioural/emotional problems)**			

**Child Characteristics**			

Male	1.3 (0.8, 2.1)	1.2 (0.7, 2.0)	0.464

**Maternal Characteristics**			

Poor parenting morale, at 3 years post partum	1.9 (1.0, 3.5)	1.9 (1.0, 3.5)	0.044

History of abuse, at pregnancy	1.9 (1.1, 3.4)	1.9 (1.1, 3.4)	0.023

**Table 5:** Predicted probability for scoring at risk of developmental problems on the PEDS, from the multinomial logistic regression model

Child Characteristics	Maternal Characteristics		Predicted Probability of Screening in Each PEDS Path			
**Gender**	**History of Abuse, at pregnancy**	**Parenting Morale, at 3 Years Post Partum**	**Path A** **(high risk for developmental problems)**	**Path B** **(moderate risk for developmental problems)**	**Path C** **(elevated risk for behavioural/emotional problems)**	**Path E** **(low risk for problems)**

Boy	Yes	Poor	0.35	0.35	0.18	0.12

Boy	No	Poor	0.27	0.33	0.18	0.22

Girl	Yes	Poor	0.24	0.34	0.24	0.18

Boy	Yes	Good	0.18	0.39	0.20	0.23

Girl	No	Poor	0.17	0.29	0.22	0.32

Boy	No	Good	0.13	0.32	0.17	0.39

Girl	Yes	Good	0.11	0.33	0.23	0.33

Girl	No	Good	0.07	0.25	0.18	0.49

## Discussion

This study indicates a direct relationship between the well being of mothers and the development of their children at school entry [14]. Maternal history of abuse reported during the prenatal period and past parenting morale were independent predictors of high risk for childhood developmental problems at school entry. Mothers of children at high risk for developmental problems were also more likely to have lower scores in measures of current mental health and parenting morale, as well as current and past social support.

This study and the previous follow-up study at three years suggests that the direct relationship between the well-being of mothers and the development of their children begins in the early years and persists through to school entry [14]. These findings suggests that children may not 'rebound' from early threats to development that occur before age 3 simply through maturity, independence or through increased interactions outside the home. This work highlights the critical importance of maternal well being and parenting morale in child development, and emphasizes that these can be unrelated to economic security and maternal education.

In particular, children of mothers who reported a history of abuse while pregnant were more than twice as likely to be at risk for developmental problems at three years and at five years [14]. These findings suggest that identifying women with a history of abuse may be one way to identify women whose children are at higher risk of developmental problems. Based on the findings from this study, identification of these women could take place as early as the prenatal period. However, further studies are required to better understand how we can identify and intervene with women in the prenatal period to remediate barriers to optimal parenting and child development outcomes [44-46]. Nevertheless, the finding that about a third of this population reported a history of abuse speaks to the potentially pervasive experience of abuse across all socio-economic domains and highlights opportunities for improved access to services and supports to address past abuse.

It is important to note that maternal history of abuse, poor parenting morale, and poor maternal social support were all associated with highest risk of developmental problems (Path A) at school entry in the bivariate analysis, but maternal history of abuse and poor parenting morale were more predictive in the logistic regression model of a child screening in Path A when accounting for other factors. This may be due to the relationship among these variables. Past research has demonstrated that women in abusive situations often have poor social support, resulting from the social systems that create an environment conducive to abuse or the social isolation imposed on women by those who abuse them [46,47]. Women who report a history of abuse, regardless of current socio-demographic circumstances, may be less willing or able to engage in supportive social relationships due to their view of self or others [46].

Children at high risk for developmental problems were more likely to have mothers who had low parenting morale when their children were three years old. It may be that mothers with low parenting morale are particularly sensitive to difficulties that may be emerging in their children, or that a child's emerging developmental difficulties and a mother's parenting morale influence each other in a reciprocal fashion. Considering this, support to parents that address sense of competence and morale may positively impact parental well being and child development.

In this study, 15% of children screened at high risk of developmental problems, similar to the proportion found when this cohort of children was three years of age. Although most children had had routine medical checkups, only about half of those who screened at high risk of developmental problems based on parents' concerns had been previously referred for assessments or interventions. The common practice among physicians for developmental surveillance is subjective clinical observation which identifies only 30 - 50% of children with developmental delay [48,49]. Physicians' developmental screening often focuses on sensory deficits, with less attention to developmental and behavioural/emotional problems. Reliable screening approaches would greatly improve the rate of identification [50]. Finding solutions for providing accessible, valid and reliable identification of developmental and behavioural/emotional problems with appropriate interventions for children and families at risk would benefit all of society.

Although the women most likely to participate in the study had high levels of education and household incomes, the sample aligns with the income level of three-quarters of all Canadian families with kids under six years of age and the educational attainment of three-quarters of Canadian women giving birth [33,51]. The results then are generalizable to the majority of Canadian families with children under six. Furthermore, 15% of children screened at highest risk of developmental problems, as would be anticipated in a population based setting.

Despite our attempts to follow-up women in the study, mothers who were younger, had lower education and income, had poor physical health, were single or divorced, and smoked were more likely to be unreachable or not respond to follow-up attempts. Thus, these findings cannot be generalized to this more vulnerable population. In other research, these factors have been associated with increased risk for poor infant outcomes and child developmental problems, and consequently, the results potentially underestimate the proportion of the total population of preschool children at risk of problems [10,12-14,19,52-54].

It is important to consider that the factors associated with women who were not retained in the cohort (young maternal age, low education and income, poor physical health, being single, and smoking) are similar to the characteristics of women who are difficult to retain in studies and in longitudinal research [55]. These factors may signal complex health, lifestyle, and social issues that these women face which make it difficult to retain them [56]. Also, the original study was a community based study that was not initially designed to be a longitudinal follow-up study. Thus, traditional strategies to retain women (e.g. incentives, changes of address cards, and routine follow-up) were not implemented immediately after the first study. However, retention strategies were implemented between the follow-up study at 3 years and at 5 years (e.g. routine contact, asking women to inform us of an upcoming change in contact information). In all three studies, the participation rates were over 60%, and the women not retained in the cohort appear to be similar among all three studies.

Because this sample included few women who had a household income less than $40,000 and none who had less than a high school education, the current study did not find differences in developmental risk by education or income level. Nevertheless, this study raises the consideration that although children may not be at risk for developmental problems due to maternal education or family income, other factors may place children at risk for developmental problems. It is noteworthy that the women in this study would be commonly defined as a "low risk" population considering their demographic information. However, one in four of these mothers had mental health issues and almost one in three had a history of abuse, which in turn may have placed their children at risk for developmental problems.

Notably, the PEDS is not diagnostic, but is rather a screening tool based on parent report of concerns. It assigns a level of risk of developmental problems based on those concerns. Thus, children who are identified as being at high risk for developmental problems may not actually be encountering developmental problems. Similarly, children whose parents do not express concerns about development may be delayed or at risk of behavioural/emotional problems. This possibility for misclassification by the PEDS must be considered when interpreting the regression model in this paper, yet the magnitude of the odds ratios and significance and consistency of the findings related to the impact of maternal factors suggest a notable relationship to child development [14,18-20,25,28,52-54].

## Conclusions

This study and the previous follow-up study indicate that the well-being of mothers is associated with developmental risk in children through the preschool years and at school entry. There is a consistent influence of maternal well-being on infant and child development, and an opportunity to better understand how women's well-being during pregnancy and early childhood can be optimized. Identifying women with a history of abuse and determining what strategies are needed to support their well-being and parenting has the potential to improve the life course trajectory for children and families. Thus, ongoing research and evaluation of interventions that enhance parenting and early assessment of child development is warranted. Failure to intervene when risk can be identified may limit efforts to optimize child development by school entry.

## Competing interests

The authors declare that they have no competing interests.

## Authors' contributions

SCT conceived and designed the study, contributed to the interpretation of data, and drafted the manuscript. JES carried out the analysis of the data, contributed to the interpretation of data, and revised the manuscript for important intellectual content. KB and SL contributed substantially to the interpretation of data and revised the manuscript for important intellectual content. DWJ coordinated the research study and revised the manuscript for important intellectual content. All authors read and approved the final manuscript.

## Pre-publication history

The pre-publication history for this paper can be accessed here:

http://www.biomedcentral.com/1471-2431/10/19/prepub

## Supplementary Material

Additional file 1 Questionnaire used at 5 year follow-up with participants from the Community Perinatal Care Study cohort Click here for file
