# Supplementary material for: Maternal well-being and its association to risk of developmental problems in children at school entry
Source: BMC Pediatr. 2010 Mar 25;10:19. doi: 10.1186/1471-2431-10-19 (PMC2858134; doi:10.1186/1471-2431-10-19)
Supplement: Additional file 1 — Questionnaire used at 5 year follow-up with participants from the Community Perinatal Care Study cohort [file 1471-2431-10-19-S1.PDF]

SECTION 1. PARENTING SUPPORTS AND RESOURCES

This section asks questions about parenting, community resources, and parenting supports and resources.

1. In the PAST YEAR, have you or your child (aged 4-6) used or attended any of the following? **Please fill in (●) all that apply to you**

- ○ Health Link (Calgary Health Region Telephone Information Line)(943-LINK or 943-5465)
- ○ 211 (City of Calgary/United Way Telephone Information-Referral Line)
- ○ Parent Link Centre(s) (an organization that provides parenting information at sites across Calgary and Alberta)
- ○ Your local community Health Centre (Calgary Health Region, community health sites NOT hospitals or doctor's offices)
- ○ Child care facilities
- ○ Drop in centres (locations where you can leave your child for a short time to run errands etc...)
- ○ Preschool or Play School Programs
- ○ Calgary's Child (free parenting magazine/newspaper)
- ○ A local Immigrant Serving Group
- ○ Families Matter Parenting and Family Programs
- ○ Boys and Girls Club
- ○ "Growing Miracles" parenting resource book
- ○ Children's music classes
- ○ Children's arts and crafts classes
- ○ A children's theatre or acting group
- ○ Informal mom's and tot's groups
- ○ Children's sport's groups (e.g. t-ball, soccer, hockey, karate)
- ○ Child swimming lessons
- ○ Your local library
- ○ A story time meeting in your community
- ○ A local fitness, recreation, or leisure centre (e.g. wave pool, wall climbing, floor hockey) (BY YOURSELF)
- ○ A local fitness, recreation, or leisure centre (e.g. wave pool, wall climbing, floor hockey) (USED BY YOUR CHILD)
- ○ Sparks or Beavers groups
- ○ A local church or spiritual leader, mentor or organization
- ○ A parenting group on the internet
- ○ Calgary Learning Centre
- ○ Family Literacy Programs
- ○ Parenting classes: If yes: What did you want from the parenting classes? (please fill in all that apply)
  - ○ What normal child development is
  - ○ How to help your child learn
  - ○ Managing family routines
  - ○ Managing your child's behaviour
  - ○ Parenting resources and supports
  - ○ To meet other parents
  - ○ Other: please specify: ______________________________

- ○ My child or I used or attended a group or resource not specified above (please specify):

____

SECTION 2. HEALTH CARE

1. Has your child (aged 4-6) gone to see a doctor for a routine physical health exam **in the past year**? [In other words, has he/she gone to a doctor to check his/her general health?]

- ○ Yes
- ○ No
- ○ Don't know

2. **In the past year** has a doctor or health worker recommended that your child (aged 4-6) go to any of the following: **[Please fill in (●) all that apply]**

(NOTE: Some examples of health workers - nurses, doctors, psychologist, occupational therapist)

- ○ An "early intervention" program?
- ○ A speech and language pathologist (someone who helps with language development, speech correction, swallowing, speech exercises if any speech problems)?
- ○ A child developmental pediatrician (i.e. pediatrician specializing in development)?
- ○ A psychologist?
- ○ A physiotherapist (helps with exercises or uses electrical currents for care of physical injury or strain)?
- ○ A dietician?
- ○ None

3. **In the past year** has your child (aged 4-6) had his/her vision or eyes checked (i.e. for sight not infection)?

- ○ Yes
- ○ No
- ○ Don't know

4. **In the past year** has your child (aged 4-6) had his/her hearing tested?

- ○ Yes
- ○ No
- ○ Don't know

5. Has your child (aged 4-6) seen a dentist **in the past year**?

- ○ Yes
- ○ No
- ○ Don't know

SECTION 3. YOUR CHILD'S LEARNING, DEVELOPMENT, BEHAVIOUR

1. Now we would like to ask about how your child (aged 4-6) is doing. Please tell us about any concerns you have with your child's learning, development and behaviour.

2. Do you have any concerns about how your child talks and makes speech sounds?
   - O No O Yes O A little

   Comments:

3. Do you have any concerns about how your child understands what you say?
   - O No O Yes O A little

   Comments:

4. Do you have any concerns about how your child uses his/her hands and fingers to do things?
   - O No O Yes O A little

   Comments:

5. Do you have any concerns about how your child uses his/her arms and/or legs?
   - O No O Yes O A little

   Comments:

6. Do you have any concerns about how your child behaves?

○ No ○ Yes ○ A little

Comments: ______

7. Do you have any concerns about how your child gets along with others?

○ No ○ Yes ○ A little

Comments: ______

8. Do you have any concerns about how your child is learning to do things for him/herself?

○ No ○ Yes ○ A little

Comments: ______

9 Do you have any concerns about how your child is learning preschool or school skills?

○ No ○ Yes ○ A little

Comments: ______

10. Please list any other concerns:

SECTION 4. ABOUT YOUR CHILD...CONTINUED

The following are statements that could describe your child (aged 4-6). Please tell us how well each of the statements actually does describe your child.

1. Your child can accept things not going his/her way.

   Not at all ○ A little ○ Moderately well ○ Well ○ Very well ○

2. Your child copes well with failure.

   Not at all ○ A little ○ Moderately well ○ Well ○ Very well ○

3. Your child thinks before acting.

   Not at all ○ A little ○ Moderately well ○ Well ○ Very well ○

4. Your child resolves problems with friends or brothers and sisters on his/her own.

   Not at all ○ A little ○ Moderately well ○ Well ○ Very well ○

5. Your child can calm down when excited or all wound up.

   Not at all ○ A little ○ Moderately well ○ Well ○ Very well ○

6. Your child does what he or she is told to do.

   Not at all ○ A little ○ Moderately well ○ Well ○ Very well ○

7. Your child is very good at understanding other people's feelings.

   Not at all ○ A little ○ Moderately well ○ Well ○ Very well ○

8. Your child controls his/her temper when there is a disagreement.

   Not at all ○ A little ○ Moderately well ○ Well ○ Very well ○

9. Your child shares things with others.

   Not at all ○ A little ○ Moderately well ○ Well ○ Very well ○

10. Your child is helpful to others.

    Not at all ○ A little ○ Moderately well ○ Well ○ Very well ○

11. Your child listens to other's points of view.

    Not at all ○ A little ○ Moderately well ○ Well ○ Very well ○

12. Your child can give suggestions and opinions without being bossy.

    Not at all ○ A little ○ Moderately well ○ Well ○ Very well ○

SECTION 5. YOUR HEALTH

This section asks for your views about <u>your</u> health. Please fill in (●) one answer for each question.

1. Overall how would you rate your health during the **<u>past 4 weeks</u>**?

   Excellent ○ Very good ○ Good ○ Fair ○ Poor ○ Very poor ○

2. During the **<u>past 4 weeks,</u>** how much did physical health problems limit your usual physical activities (such as walking or climbing stairs)?

   Not at all ○ Very little ○ Somewhat ○ Quite a lot ○ Could not do physical activities ○

3. During the **<u>past 4 weeks,</u>** how much difficutly did you have doing your daily work, both at home and away from home, because of your physical health?

   Not at all ○ Very little ○ Somewhat ○ Quite a lot ○ Could not do physical activities ○

4. How much bodily pain have you had during the **<u>past 4 weeks</u>**?

   None ○ Very mild ○ Mild ○ Moderate ○ Severe ○ Very severe ○

5. During the **<u>past 4 weeks,</u>** how much energy did you have?

   Very much ○ Quite a lot ○ Some ○ A little ○ None ○

6. During the **<u>past 4 weeks,</u>** how much did your physical health or emotional problems limit your usual social activities with family or friends?

   Not at all ○ Very little ○ Somewhat ○ Quite a lot ○ Could not do social activities ○

7. During the **<u>past 4 weeks,</u>** how much have you been bothered by **<u>emotional problems</u>** (such as feeling anxious, depressed or irritable)?

   Not at all ○ Slightly ○ Moderately ○ Quite a lot ○ Extremely ○

8. During the **<u>past 4 weeks,</u>** how much did personal or emotional problems keep you from doing your usual work, school or other daily activities?

   Not at all ○ Very little ○ Somewhat ○ Quite a lot ○ Could not do daily activities ○

SECTION 5. YOUR HEALTH

The following questions ask how you feel in your daily life as a parent. (please fill in (●) one answer)

When you think of your daily life as a parent how often do you feel............

1. Optimistic (looking forward to the future and current life)?

   Not at all ○ Rarely ○ Sometimes ○ Often ○ Very often ○

2. Worried?

   Not at all ○ Rarely ○ Sometimes ○ Often ○ Very often ○

3. Contented?

   Not at all ○ Rarely ○ Sometimes ○ Often ○ Very often ○

4. Frustrated?

   Not at all ○ Rarely ○ Sometimes ○ Often ○ Very often ○

5. Satisfied?

   Not at all ○ Rarely ○ Sometimes ○ Often ○ Very often ○

6. Happy?

   Not at all ○ Rarely ○ Sometimes ○ Often ○ Very often ○

7. Stressed?

   Not at all ○ Rarely ○ Sometimes ○ Often ○ Very often ○

8. Lonely?

   Not at all ○ Rarely ○ Sometimes ○ Often ○ Very often ○

9. Exhausted?

   Not at all ○ Rarely ○ Sometimes ○ Often ○ Very often ○

10. Guilty?

   Not at all ○ Rarely ○ Sometimes ○ Often ○ Very often ○

Please indicate how much you agree or disagree to the following questions.

1. The problems of taking care of a child are easy to solve once you know how your actions affect your child, an understanding I have acquired.

   Strongly Agree ○ Agree ○ Slightly Agree ○ Slightly Disagree ○ Disagree ○ Strongly Disagree ○

2. I meet my own personal expectations for expertise in caring for my child.

Strongly Agree ○ Agree ○ Slightly Agree ○ Slightly Disagree ○ Disagree ○ Strongly Disagree ○

3. I would make a fine model for a new parent to follow in order to learn what they would need to know to be a good parent.

Strongly Agree ○ Agree ○ Slightly Agree ○ Slightly Disagree ○ Disagree ○ Strongly Disagree ○

4. Being a parent is manageable, and my problems are easily solved.

Strongly Agree ○ Agree ○ Slightly Agree ○ Slightly Disagree ○ Disagree ○ Strongly Disagree ○

5. If anyone can find the answer to what is troubling my child, I am the one.

Strongly Agree ○ Agree ○ Slightly Agree ○ Slightly Disagree ○ Disagree ○ Strongly Disagree ○

6. A difficult problem in being a parent is not knowing whether you're doing a good job or a bad one.

Strongly Agree ○ Agree ○ Slightly Agree ○ Slightly Disagree ○ Disagree ○ Strongly Disagree ○

7. Considering how long I've been a parent, I feel thoroughly familiar with this role.

Strongly Agree ○ Agree ○ Slightly Agree ○ Slightly Disagree ○ Disagree ○ Strongly Disagree ○

8. I honestly believe I have all the skills necessary to be a good parent to my child.

Strongly Agree ○ Agree ○ Slightly Agree ○ Slightly Disagree ○ Disagree ○ Strongly Disagree ○

9. Even though being a parent could be rewarding, I am frustrated now while my child is at his/her present age.

Strongly Agree ○ Agree ○ Slightly Agree ○ Slightly Disagree ○ Disagree ○ Strongly Disagree ○

10. I do not know why it is, but sometimes when I'm supposed to be in control, I feel more like the one being manipulated.

Strongly Agree ○ Agree ○ Slightly Agree ○ Slightly Disagree ○ Disagree ○ Strongly Disagree ○

11. My mother/father was better prepared to be a good mother/father than I am.

Strongly Agree ○ Agree ○ Slightly Agree ○ Slightly Disagree ○ Disagree ○ Strongly Disagree ○

12. Sometimes I feel like I'm not getting anything done.

Strongly Agree ○ Agree ○ Slightly Agree ○ Slightly Disagree ○ Disagree ○ Strongly Disagree ○

13. I go to bed the same way I wake up in the morning -- feeling I have not accomplished a whole lot.

Strongly Agree ○ Agree ○ Slightly Agree ○ Slightly Disagree ○ Disagree ○ Strongly Disagree ○

14. My talents and interests are in other areas, not in being a parent.

Strongly Agree ○ Agree ○ Slightly Agree ○ Slightly Disagree ○ Disagree ○ Strongly Disagree ○

15. If being a parent were only more interesting, I would be motivated to do a better job.

Strongly Agree ○ Agree ○ Slightly Agree ○ Slightly Disagree ○ Disagree ○ Strongly Disagree ○

16. Being a parent makes me tense and anxious.

Strongly Agree ○ Agree ○ Slightly Agree ○ Slightly Disagree ○ Disagree ○ Strongly Disagree ○

Next are some questions about the support that is available to you.

1. About how many close friends and close relatives do you have (people you feel at ease with and can talk to about what is on your mind)?___________(write in number of close friends and relatives)

2. People sometimes look to others for companionship, assistance, or other types of support.

How often is each of the following kinds of support available to you if you need it?

Fill in only one circle for each line

	None of the Time	A little of the Time	Some of the Time	Most of the Time	All of the Time
Someone to help you if you were confined to bed	○	○	○	○	○
Someone you can count on to listen to you when you need to talk	○	○	○	○	○
Someone to give you good advice about a crisis	○	○	○	○	○
Someone to take you to the doctor if you needed it	○	○	○	○	○
Someone who shows you love and affection	○	○	○	○	○
Someone to have a good time with	○	○	○	○	○
Someone to give you information to help you understand a situation	○	○	○	○	○
Someone to confide in or talk to about yourself or your problems	○	○	○	○	○
Someone who hugs you	○	○	○	○	○
Someone to get together with for relaxation	○	○	○	○	○
Someone to prepare your meals if you were unable to do it yourself	○	○	○	○	○
Someone whose advice you really want	○	○	○	○	○
Someone to do things with to help you get your mind off of things	○	○	○	○	○
Someone to help with daily chores if you were sick	○	○	○	○	○
Someone to share your most private worries and fears with	○	○	○	○	○
Someone to turn to for suggestions about how to deal with a personal problem	○	○	○	○	○
Someone to do something enjoyable with	○	○	○	○	○
Someone who understands your problems	○	○	○	○	○
Someone to love and make you feel wanted	○	○	○	○	○

3. How would you describe the social support that you receive (e.g. from a partner, friend, family)?
For example, if you have a car and it breaks down, you need to talk about a decision you have to make, you need someone to listen to you, you need help moving, or you need someone to take care of your child, is there someone who can help you or talk with you?

	Excellent	Good	Fair	Poor	Terrible
The social support I receive is...	O	O	O	O	O

4. On a scale of 1 (no support) to 10 (fully supported), please rate how satisfied you are with the support you receive from your partner. **Please fill in (●) one number only.**

No Support				Average Support					Fully Supported	I do not have a partner
O	O	O	O	O	O	O	O	O	O	O
1	2	3	4	5	6	7	8	9	10	NA

5. On a scale of 1 (no support) to 10 (fully supported), please rate how satisfied you are with the support you receive from your family.. **Please fill in (●) one number only.**

No Support				Average Support					Fully Supported	I do not have a family
O	O	O	O	O	O	O	O	O	O	O
1	2	3	4	5	6	7	8	9	10	NA

6. On a scale of 1 (no support) to 10 (fully supported), please rate how satisfied you are with the support you receive from your friends. **Please fill in (●) one number only.**

No Support				Average Support					Fully Supported	I do not have a friends
O	O	O	O	O	O	O	O	O	O	O
1	2	3	4	5	6	7	8	9	10	NA

Note: The last series of questions were about feelings people commonly have, and we recognize that answering them may cause some emotional discomfort. Should that be the case, counseling resources are available to you by calling the Calgary Counseling Centre at 403-265-4980, the Calgary Health Link at 403-943-5465, Families Matter at 403-205-5178, the Distress Centre at 403-266-1605, or by talking to your family doctor.

SECTION 6. BACKGROUND QUESTIONS

Finally, we would like to ask you background questions about yourself. Your answers are confidential. We use this information to compare groups of people not specific individuals.

1. What is your current marital status? At present are you:

   - ○ Single (never married)
   - ○ Married
   - ○ Common Law/Living with Partner/Living as Married
   - ○ Divorced
   - ○ Separated
   - ○ Widowed

2. Which of the following best describes your **MAIN** activity (please fill in (●) one answer only)? Are you mainly...

   - ○ Working at a job or business (either part-time, full-time, or casual)
   - ○ A homemaker
   - ○ Looking for work
   - ○ On paid maternity leave
   - ○ A student
   - ○ Other, specify: ______________________________

3. What is the total income, **before taxes and deductions**, of all household members from all sources in the past 12 months (your best guess is ok)? **Was the total household income:**

   - ○ Less than $20,000
   - ○ $20,000 - $39,999
   - ○ $40,000 - $59,999
   - ○ $60,000 - $79,999
   - ○ $80,000 - $99,999
   - ○ $100,000 - $119,999
   - ○ $120,000 - $139,999
   - ○ $140,000 - $159,999
   - ○ $160,000 or more
   - ○ I prefer not to answer this question

4. How many times have you moved in the past 2 years?__________times

5. What is the highest level of schooling you have completed?

- ○ Grade School
- ○ High School
- ○ College or technical school
- ○ University Degree
- ○ Post-Graduate Degree (e.g. PhD, MD, MA, MSc)

6. Please enter today's date:Day_________Month_________Year_________

7. Can we contact you if we have questions about this survey?

- ○ Yes: please tell us your telephone number: (   ) ____ ______________________ (optional)
- ○ No

8. Can we contact you to invite you to participate in future research projects or programs?

- ○ Yes: please tell us your telephone number: (   ) ______________________ (optional)
- ○ No

or email:____ if available (optional)

9. When you previously participated in the Community Perinatal Care Study you gave us permission to access the health records for your pregnancy. Do you give us permission to access the health records for your child's health? We only look at this information in group format, and names or any personal identifying information is not included in the health data in the Calgary Health Region or Alberta Health

- ○ Yes: please provide your Alberta Health Care card number:______________________

  your Child's Alberta Health card number:______________________

  (note: you do not need to provide this information in order to participate in this study)
- ○ No

That concludes the questionnaire: **Thank you so much for your time!** It's people like you who help provide the best health care possible to children and women. Please return the questionnaire in the envelope that is provided.

If you are interested in a copy of the results please provide us with your e-mail:______________________
